# Proton Transport in Cancer Cells: The Role of Carbonic Anhydrases

**DOI:** 10.3390/ijms22063171

**Published:** 2021-03-20

**Authors:** Holger M. Becker, Joachim W. Deitmer

**Affiliations:** 1 Zoology and Animal Physiology, Institute of Zoology, TU Dresden, D-01217 Dresden, Germany; 2 Department of Biology, University of Kaiserslautern, D-67653 Kaiserslautern, Germany; deitmer@biologie.uni-kl.de

**Keywords:** proton antenna, transport metabolon, hypoxia, cancer cell metabolism, pH regulation

## Abstract

Intra- and extracellular pH regulation is a pivotal function of all cells and tissues. Net outward transport of H^+^ is a prerequisite for normal physiological function, since a number of intracellular processes, such as metabolism and energy supply, produce acid. In tumor tissues, distorted pH regulation results in extracellular acidification and the formation of a hostile environment in which cancer cells can outcompete healthy local host cells. Cancer cells employ a variety of H^+^/HCO_3_^−^-coupled transporters in combination with intra- and extracellular carbonic anhydrase (CA) isoforms, to alter intra- and extracellular pH to values that promote tumor progression. Many of the transporters could closely associate to CAs, to form a protein complex coined “transport metabolon”. While transport metabolons built with HCO_3_^−^-coupled transporters require CA catalytic activity, transport metabolons with monocarboxylate transporters (MCTs) operate independently from CA catalytic function. In this article, we assess some of the processes and functions of CAs for tumor pH regulation and discuss the role of intra- and extracellular pH regulation for cancer pathogenesis and therapeutic intervention.

## 1. Introduction

Cancer cells, especially those located in a hypoxic microenvironment, often display a significant increase in glycolysis, as compared to healthy cells. This deregulation in cellular energetics has been considered as a hallmark of cancer [1]. Metabolic reprogramming of cancer cells can be elicited by local, intermitted or long term, hypoxia, which arises from unrestrained cell proliferation and insufficient or chaotic vascularization of the tumor mass [2]. However, tumors display considerable metabolic heterogeneity. Therefore, cancer cells can also produce their energy not only by glycolysis, but also from oxidation in the tricarboxylic acid (TCA) cycle [3,4,5,6]. Excessive production of metabolic acids, either in the form of CO_2_ or lactate and protons, together with a distorted pH regulation, results in extracellular acidification and the formation of a hostile environment, in which cancer cells, which tolerate considerably low pH_e_ values, can outcompete healthy local host cells [7,8,9,10,11]. Besides killing adjacent host cells, the low pH_e_ facilitates cell migration and the formation of metastasis, e.g., by pH-dependent modulation of integrin-mediated cell-matrix adhesion and degradation of the extracellular matrix [11,12,13,14,15]. Furthermore, the acid microenvironment supports immune escape of cancer cells by inhibiting chemotaxis of immune cells [16] and T-cell activation [17]. Metabolic reprogramming and modifications in the pH-regulation lead to a small intracellular alkalization, which has a profound impact on the cells’ physiology. High pH_i_ promotes cell proliferation and reduces apoptosis, and, in addition, drives cell migration, invasion and formation of metastasis [12,18,19,20,21,22,23]. Therefore, changes of intra- and extracellular pH in cancer cells can be considered as an evolutionary strategy, by which cancer cells create a tumor-permissive microenvironment, in which they can grow and outcompete non-cancer cells. Thereby, the acidic tumor environment favors cancer somatic evolution [9,11,24,25,26]. Regulation of intracellular and extracellular tumor pH is governed by the concerted interplay between cytosolic and exofacial carbonic anhydrases and acid/base transporters, the expression and activity of which are often modified during tumorigenesis.

## 2. Carbonic Anhydrases in Cancer Cells

Out of the 15 human CA isoforms, CAIX and CAXII have received most attention in cancer tissue, both as diagnostic markers and potential drug targets. CAIX and CAXII can be highly overexpressed in many tumors and are, therefore, often regarded as tumor-associated carbonic anhydrases [10,27]. While CAXII is highly expressed in many healthy tissues, including kidney, prostate, pancreas, intestine and lymphocytes, expression of CAIX in healthy tissue is believed to be restricted largely to stomach and gut epithelial tissues [28,29,30,31]. Due to this limited expression in healthy cells and the strong upregulation in many aggressive tumors, the CAIX isoform is the preferred isoform for pharmacological intervention. Expression of CAIX is controlled by the hypoxia-inducible factor HIF1α. Therefore, the enzyme is often upregulated in hypoxic tumor regions [32]. Under normoxia HIF1α is constantly hydroxylated at conserved proline residues and marked for ubiquination and proteosomal degradation by the von Hippel–Lindau tumor suppressor (pVHL). Hypoxia stabilizes HIF1α which could form a complex with HIF1β in the nucleus. The HIF1 complex binds to the hypoxia responsive element (HRE) in the *CA9* promotor to induce gene transcription and increase the expression level of CAIX [32,33]. Under chronic and mild hypoxia, CAIX expression can also be regulated by components of the mitogen-activated protein kinase (MAPK) pathway [34,35]. Furthermore, expression of CAIX can be induced by inactivation mutations of the von Hippel–Lindau tumor suppressor (VHL) gene, which leads to constitutive activation of HIF [36,37]. Expression of CAIX is often associated with chemoresistance and an overall poor prognosis in most cancers [38,39,40,41]. In contrast to CAIX, overexpression of CAXII has been linked to both good and bad tumor prognosis. In colorectal and kidney cancer as well as in oral squamous carcinoma, CAXII was found to correlate with poor prognosis [29,42]. In breast, lung and cervical cancer, however, CAXII was shown to correlate with good outcome [43,44,45]. Even though CAIX and CAXII are considered to be the most important CA isoforms in development of tumors, other CA isoform may also play a role in cancer progression. CA isoform I, for example, contributes to microcalcification, tumorigenesis and migration of breast cancer cells [46]. Like CAIX and CAXII, expression of CAII is upregulated in a variety of cancers. However, in the majority of the investigated tumors, a down-regulation of CAII is associated with poor prognosis [47,48,49]. For a comprehensive review about CA isoforms in cancer see [50].

**Figure 1:**
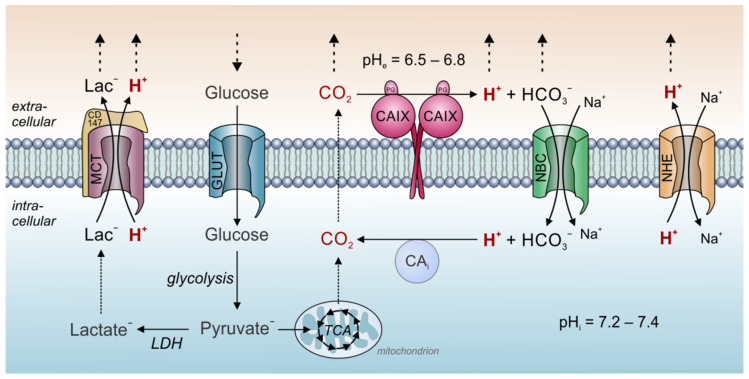
Tumor pH regulation by carbonic anhydrase and acid/base transporters. In tumor cells, metabolic acids are produced primarily by glycolysis and subsequent hydrolysis of ATP (lactate^−^ + H^+^), and mitochondrial respiration (CO_2_). At the outer face of the cell membrane, CO_2_ is hydrated by CAIX to form HCO_3_^−^ and H^+^. This allows the parallel diffusion of all three ion species through the extracellular space accelerating CO_2_ removal to the blood capillary. Furthermore, the hydration of CO_2_ by CAIX sets extracellular pH to a more acidic value. A fraction of the HCO_3_^−^ is reimported into the cell by Na^+^/HCO_3_^−^ cotransporters (NBC). In the cytosol, HCO_3_^−^ reacts with H^+^ to form new CO_2_, which can leave the cell by diffusion. Thereby, NBC supports the venting of H^+^ from the cell and contributes to cytosolic pH regulation. Protons are also removed from the cell by Na^+^/H^+^ exchangers (NHE) and in cotransport with lactate by monocarboxylate transporters (MCT). In the figure, solid arrows symbolize catalytic reactions or ion transport. Dotted arrows symbolize ion diffusion.

Carbonic anhydrases are of fundamental importance for dynamics of both intracellular and extracellular pH in tumors. Thereby, a central role is attributed to CAIX, which was suggested to function as a “pH-stat”, which sets tumor pH_e_ to a tightly controlled acidic value. Tumors display considerable metabolic heterogeneity and produce a considerable fraction of their energy not only by glycolysis, but also from oxidation in the TCA (for review see [51,52,53]). Therefore, CO_2_ is a significant source of metabolic acid production also in cancer cells [54]. However, the mere release of metabolic acids alone does not suffice to fully describe the low pH_e_ values found in solid tumors. First evidence for a role of CAIX in pH_e_ control was provided by Svastova and colleagues [55], who showed that ectopic expression of CAIX in hypoxic MDCK canine kidney epithelial cells in culture leads to an acidification of the extracellular medium. Furthermore, they could show that inhibition of CAIX catalytic activity as well as overexpression of a catalytically inactive CAIX mutant reduced extracellular acidification in hypoxic HeLa cells [55]. A later study by Switach et al [56] showed that expression of CAIX in spheroids of HCT116 human colon carcinoma cells results in a higher pH_i_ (6.6 with CAIX vs. 6.3 without CAIX) and a more acidic pH_e_ (6.6 vs. 6.9). The results were confirmed by a study in HCT116 tumor xenografts, which showed that expression of exofacial CAIX results in a slight extracellular acidification (6.71 vs. 6.86) without changing pH_i_ [57]. The ability of CAIX to set pH_e_ precisely to these values might arise from the enzyme’ unique catalytic kinetics. Measurements of CAIX catalytic activity with gas-analysis mass spectrometry revealed that at a pH of 7.4, the enzyme’s rate constant for hydration was faster than for dehydration [58]. At pH values below 6.8, however, the rate of dehydration exceeded the hydration rate. For pH 6.8, the rate constants for hydration and dehydration were essentially the same [58]. CAIX displays an apparent p*K* of 6.84 and is inhibited at lower pH values [59]. Therefore, a low pH_e_ could limit further H^+^-production by CAIX in the extracellular space. In other words, at pH values below 6.8, CAIX favors the dehydration reaction, while at pH values above 6.8 the hydration reaction is preferred [55]. Taken together, these observations indicate that CAIX functions as a pH-stat that sets tumor pH_e_ to an acidic value of around 6.8 [55,56,57,58,59]. This more acidic pH_e_ value can be regarded as an evolutionary strategy of cancer cells (“niche engineering”) to create an environment that promotes tumor growth and tumor invasion. In addition, hydrolysis of cell-derived CO_2_ by CAIX, allows the parallel diffusion of CO_2_, HCO_3_^−^ and H^+^ to the blood capillaries, thereby speeding up CO_2_ venting from the cell [56] (Figure 1).

Besides controlling acidity of the extracellular environment, CAs have also been attributed a central function in the regulation of intracellular pH. Heterologous expression of exofacial CAIX in spheroids of RT112 bladder carcinoma cells induces a near uniform pH_i_, while spheroids of WT RT112 cells not expressing CAIX, or spheroids in which CAIX was pharmacological inhibited with acetazolamide, exhibited an acidic core [60]. The study concluded that CAIX coordinates the spatial pH_i_ spectrum by facilitating CO_2_ diffusion in the extracellular space. Interestingly, catalytic activity of intracellular CA seems to be of minor importance as compared to extracellular CA catalytic activity. Indeed, it was demonstrated that in cancer cells with high intracellular CA activity, fluctuations in the extracellular CO_2_ concentration (which can occur in poorly vascularized tumors) evoked faster and larger pH_i_ oscillations [61]. These pH_i_ oscillations increased Ca^2+^ oscillations, as well as inhibited the mTORC1 pathway, which is a common driver for tumor progression. These findings might explain why low expression of intracellular CAII is associated with good prognosis in various cancer types [47,48,49].

## 3. Acid/Base Transport Proteins in Cancer Cells

Even though CAs play a major role in acid venting from cancer cells, regulation of pH_i_ requires the constant extrusion of acid via transport proteins (Figure 1). One of the major proton extruders in mammalian cells is the Na^+^/H^+^-exchanger NHE1 (SLC9A1). Oncogene-induced overexpression of NHE1, which often occurs already at an early stage in cancer development, results in intracellular alkalinization and extracellular acidification [62]. This shift in the pH balance is considered a key driver in malignant transformation and progression of tumors [63,64]. NHE1 is further involved in cell migration and the formation of metastasis. In migrating cells, NHE1 redistributes to the protruding edge of the cell. Thereby, it contributes to the formation of a pH gradient along the cell, with an acidic pH_e_ and alkaline pH_i_ at the migrating front. While the low pH_e_ modulates local cell adhesion, higher pH_i_ values support the reorganization of the cytoskeleton and local cell swelling to drive cancer cell migration and invasion [65,66,67]. For a comprehensive review of the various roles of NHEs in tumor development and progression see [63].

Protons are also extruded from the cell via monocarboxylate transporters (MCTs), which mediate the H^+^-coupled transport of lactate and other monocarboxylates like pyruvate, ketone bodies and branched chain keto acids across the cell membrane [68,69,70,71] (Figure 1). Lactate coupled H^+^-extrusion is pivotal for hypoxic tumor cells, which show a drastic increase in glycolytic activity. The major MCT isoforms found in cancer cells are MCT1 (SLC16A1) and MCT4 (SLC16A3) [10]. Expression of MCT1 and MCT4 were found to be upregulated in many cancer types, including breast cancer [72,73], colorectal cancer [74], glioblastoma [75,76], prostate cancer [77], clear cell renal cell carcinoma [78], and lung carcinomas [79]. Interestingly, a recent study demonstrated that inhibition of MCT transport activity with the non-steroidal anti-inflammatory drug diclofenac could restrict tumor proliferation and increase the efficiency of immune checkpoint therapy [80]. 

Proton extrusion from cancer cells can also be mediated by V-type H^+^-ATPase. Although these proton pumps are usually located in intracellular vesicles in healthy cells, V-type H^+^-ATPases have been shown to relocate to the plasma membrane of several types of cancer cells, where they mediate proton extrusion [81,82].

Intracellular pH homeostasis is not only controlled by the constant export of protons, but also by the import of HCO_3_^−^ into cells. HCO_3_^−^ import is mainly mediated by the Na^+^/HCO_3_^−^ cotransporters NBCn1 (SLC4A7) and NBCe1 (SLC4A4). NBC-mediated import of HCO_3_^−^ supports CO_2_/HCO_3_^−^-dependent pH-buffering of the cytosol [83]. Indeed, NBCs, which are often overexpressed in human breast cancer tissue [64,84], have been suggested to function as the prime acid/base regulators in those cells [84]. Like NHE1, NBCe1 accumulates at the leading edge of migrating cells [85,86]. In this compartment, NBCe1 can cooperate with the Cl^−^/HCO_3_^−^ exchanger AE2 (SLC4A2) to drive cell migration. In healthy cells, AE2 functions as a HCO_3_^−^ exporter, which either protects the cell from intracellular alkalosis or mediates alkalization of the extracellular fluid [87,88]. In cancer cells, which produce and release high amounts of acid, AE2 is primarily involved in the facilitation of cell migration [8,12]. AE2 was shown to accumulate at the leading edge of migrating cancer cells, where it colocalizes with NBCe1 and NHE1 [85,86]. In this compartment, AE2 exports HCO_3_^−^ (which was imported by the NBC) in exchange for osmotically active Cl^−^ to support local water uptake via aquaporins [86]. The cooperation between NBCn1 and AE2, together with the local activity of NHE1, results in the net-import of Na^+^ and Cl^−^ to support local swelling of the lammelipodia and drive cell migration [12,85,86].

## 4. Acid/Base Transport Metabolons in Cancer Cells

Most of the acid/base transporters overexpressed in cancer cells are associated with intra and/or extracellular CAs to form a protein complex coined transport metabolon. A metabolon has been defined as a “temporary, structural-functional, supramolecular complex of sequential metabolic enzymes and cellular structural elements, in which metabolites are passed from one active site to another, without complete equilibration with the bulk cellular fluids (channeling)” [89,90,91]. Evidence for the existence of acid/base transport metabolons have been found in various cells and tissues, including erythrocytes [92,93], cardiomyocytes [94], astrocytes [95], and gastric mucosa [96]. For a complete list of transport metabolons, see Table 1 in [97]. First direct evidence for the existence of a transport metabolon in cancer cells was provided in 2012 by Svastova et al [86]. Using conventional immunocytochemistry and an in situ proximity ligation assay (PLA), the authors could show that CAIX is closely colocalized with NBCe1 and AE2 in hypoxic A549 lung carcinoma cells and SiHa squamous cell carcinoma cells, respectively. Interestingly, the colocalization between the bicarbonate transporters and CAIX was most evident in the leading edge of migrating cells [86,98]. In line with these findings, it was shown that expression of catalytic active CAIX facilitates cell migration in MDCK and hypoxic HeLa cells [86], while inhibition of CAIX catalytic activity results in impaired formation of invadopodia and degradation of the extracellular matrix [98]. These results lead to the conclusion that CAIX forms a transport metabolon with HCO_3_^−^ transporters in the leading edge of migrating cancer cells to facilitate ion transport and pH control at the protruding front of the moving cell and thus drives cancer cell migration [86,98]. CAIX does not only interact with HCO_3_^−^ transporters in cancer cells, but is also closely colocalized with the Na^+^/H^+^ exchanger NHE1 and the Na^+^/Ca^2+^ exchanger NCX1 (SLC8A1), as shown by in situ PLA in hypoxic SiHa cells [99]. Physical interaction between the three proteins was demonstrated by immune co-precipitation of NHE1 and NCX1 with CAIX in SiHa cell lysates [99]. Interestingly, silencing or pharmacologic inhibition of NCX1 diminished the ability of hypoxic cancer cells to control their pH_i_, even though NCX1 does not transport acid/base equivalents itself. NCX1, NHE1 and CAIX seem to form a protein complex which operates as a transport metabolon to extrude protons from the cells. Within this complex, CAIX supports NHE1 activity, by removing H^+^ from the extracellular site of the transporter pore, thereby stabilizing the H^+^ gradient for the NHE1. NCX1 and NHE1 create a Na^+^ short circuit which stabilizes the local Na^+^ gradient for the proton extruder [99].

Even though there is strong evidence that CAIX might form transport metabolons with acid/base transporters in cancer cells, studies which have investigated the molecular mechanism by which CAIX facilities transport activity of NHE, NBC and AE are still scarce. Coexpression of the Cl^−^/HCO_3_^−^ exchangers AE1 (SLC4A1), AE2 (SLC4A2) and AE3 (SLC4A3) with CAIX in HEK293 cells increased AE-mediated bicarbonate transport [96]. Co-immunoprecipitation of AE1, AE2 and AE3 with CAIX and truncation mutants of the enzyme revealed direct binding of the CAIX catalytic domain to the transporters [96]. In line with these findings, pull-down experiments with GST-fusion proteins demonstrated that CAIX binds to the 4th extracellular loop of the AE1 protein [94]. The same study also demonstrated binding between CAIX and the 4th extracellular loop of the Na^+^/HCO_3_^−^ cotransporter NBCe1 and showed that inhibition of CAIX catalytic activity with 6-ethoxy-2-benzothiazolesulfonamide (EZA) decreased NBCe1 transport activity in NBCe1-expressing HEK293 cells [94]. Taken together, these data indicate that CAIX binds to an extracellular moiety of the membrane acid/base transporters to catalyze the interconversion of CO_2_ and HCO_3_^−^/H^+^ in the immediate vicinity of the transporter and hence facilitates bicarbonate transport activity. These findings are in line with other studies on the physical and functional interaction between acid/base transporters and intracellular and extracellular carbonic anhydrases [100,101,102,103,104,105,106]. Taken together, these studies demonstrate that CA-mediated facilitation of acid/base transport activity requires both CA catalytic activity and direct binding of the enzyme to the transporter. However, it should also be noted that the general concept of a transport metabolon has been questioned by several studies, both with respect to the formation of a protein complex as well as to the functional significance of colocalization between transporter and enzyme [107,108,109,110]. An in-depth discussion on the various types of acid/base transport metabolons, including the controversy about this concept, is given by a number of reviews [91,97,111,112,113,114].

## 5. Non-Catalytic Transport Metabolons in Cancer Cells

In contrast to the transport metabolons described so far, monocarboxylate transporters form transport metabolons with carbonic anhydrases that operate independent from the enzyme’s catalytic activity. The existence of such a non-catalytic transport metabolon in cancer cells was first described for hypoxic breast cancer cells [115]. By measuring changes in intracellular lactate concentration with the lactate-sensitive FRET nanosensor laconic [116], the authors could show that lactate flux is significantly increased in hypoxic cancer cells expressing CAIX as compared to normoxic cells without CAIX [115]. Expression levels of MCTs, however, remained unaltered under hypoxia. Interestingly, chemical inhibition of CAIX catalytic activity did not reverse the augmentation of lactate flux in hypoxic cancer cells, while siRNA-mediated knock-down of CAIX decreased lactate flux to the level observed in normoxic cells [115]. From these data the authors concluded that CAIX facilitates lactate flux in cancer cells by a non-catalytic mechanism. This assumption was supported by measurements of the glycolytic Proton Efflux Rate (glycoPER) in the triple negative breast cancer cell line UFH-001 with a Seahorse analyzer [117]. The cells were treated with the HIF1α-stabilizing agent desferrioxamine (DFO) to simulate hypoxia. CRISPR/Cas9-mediated knockdown of CAIX resulted in a significant decrease in glycoPER, while isoform-specific inhibition of CAIX catalytic activity with three ureido-substituted benzene sulfonamides did not affect proton flux. In line with these results, the same group could demonstrate that full inhibition of CAIX catalytic activity with the imidazole-substituted benzenesulfonamide SLC-149 did not affect growth of MCF10A, UFH-001, and T47D breast cancer cells, indicating a non-catalytic function of CAIX in cancer cell proliferation [118]. An in situ PLA demonstrated close colocalization between MCT1/MCT4 and CAIX in hypoxic breast cancer cells [119]. These data indicate that CAIX forms a protein complex with MCTs to facilitate lactate transport via a mechanism that does not involve CAIX catalytic activity. The MCT1/4-CAIX metabolon was not only found in cultivated breast cancer cells, but also in tissue samples of human breast cancer patients, with an increasing amount of transport metabolons in higher grade tumors [119]. 

The mechanisms underlying this non-catalytic facilitation of lactate flux were elucidated by heterologous protein expression in *Xenopus* oocytes. Co-expression of MCT1 and MCT4 with CAIX resulted in a doubling of MCT transport activity, resembling the observations made in breast cancer cells [115]. Pull-down experiments and pH-measurements in *Xenopus* oocytes revealed that CAIX does not directly bind to the transporter itself, but to the Glu73 residue in the Ig1 domain of the MCT1/4 chaperon CD147 [119]. Interestingly, binding was mediated by His200 in the CAIX catalytic domain (also referred to His64 in the CAII nomenclature). This histidine is considered to be the central residue of the CAIX intramolecular proton shuttle, which mediates the rapid exchange of H^+^ between the enzyme’s catalytic center and the surrounding bulk solution [120]. This binding resembles the interaction between MCTs and the extracellular carbonic anhydrase CAIV. Like CAIX, CAIV binds to the Ig1 domain of the MCT1/4 chaperon CD147 and the MCT2 chaperon GP70 [121]. Binding of CAIV is mediated by human CD147-Glu73, rat CD147-Lys73, and GP70-Arg130, suggesting that the position of the binding site is conserved among the chaperons, while the identity of the amino acid can vary. In CAIV, binding is mediated by His88, the central residue of the CAIV intramolecular proton shuttle, and the analogue to CAIX-His200 [121]. These data indicate that CAIX forms a complex with MCTs by hydrogen binding to the transporter’s chaperon. In line with this, application of an antibody against the CAIX binding site of CD147 removed CAIX from the transporter-chaperon complex, as shown by in situ PLA, decreased MCT transport activity in hypoxic breast cancer cells, and inhibited glycolytic activity as well as cell proliferation [117]. These data show that the increase in lactate transport capacity, mediated by the MCT1/4-CD147-CAIX transport metabolon, is crucial to maintain a high rate of glycolysis and proliferation in hypoxic breast cancer cells.

**Figure 2:**
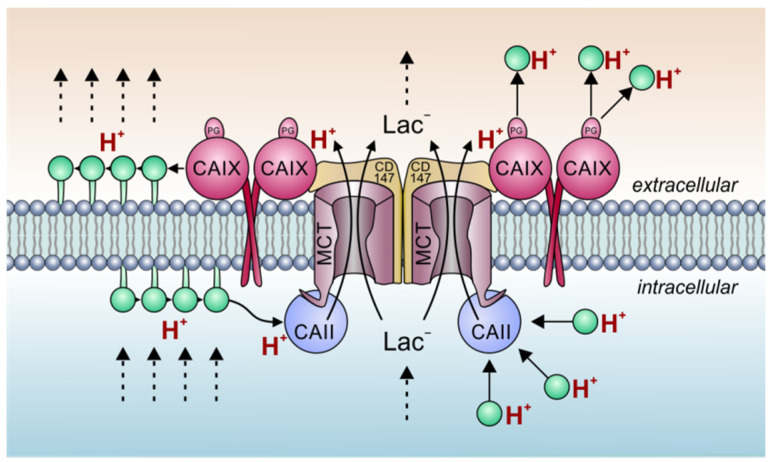
Carbonic anhydrases function as proton antennae for MCTs. Intracellular and extracellular carbonic anhydrases form a non-catalytic transport metabolon with MCT1 and MCT4. The interaction is independent of CA catalytic activity, but requires a special set of proton-collecting residues in the CA protein (CAII-Glu69/Asp72 and the CAIX-PG domain). Extracellular-facing CAIX binds to the Ig1 domain of the MCT1/4 chaperon CD147, while intracellular CAII binds to the transporter’s C-terminal tail. This binding positions the enzymes close enough to the transporter pore to establish an efficient proton shuttle between transporter and enzymes. During proton/lactate efflux, CAII collects H^+^ from surrounding protonatable residues of yet unknown identity (green circles) near or at the plasma membrane and shuttles them to the transporter. On the extracellular site, CAIX removes H^+^ from the transporter pore and shuttles them to protonatable residues at the extracellular face of the plasma membrane or in the extracellular space. This rapid exchange of H^+^ impairs the formation of proton microdomains around the transporter pore and drives the efflux of protons and lactate out of the cell. Note that both CAIX and the MCT1/4-CD147 complex exist as dimers at the cell membrane [137,138]. In the figure, dotted arrows symbolize ion diffusion. Solid arrows symbolize ion transport or proton transfer.

However, since CAIX catalytic activity is not required for the increase in MCT transport activity, the question remained as to which mechanism the transport metabolon facilitates lactate flux. Proton diffusion in an unbuffered solution is very fast (diffusion coefficient D^H^ = 1187 × 10^−7^ cm^2^ s^−1^ [122]). However, in a highly buffered solution such as the cytosol or the extracellular space, H^+^ diffusion is much slower. In rabbit ventricular myocytes, an apparent D^H^ of 3.78 × 10^−7^ cm^2^ s^−1^ was found, which is more than 300 times lower than the D^H^ in an unbuffered solution [123]. Indeed, due to the high buffering of protons, H^+^ mobility resembles the diffusion rate of mobile buffers [123]. The low H^+^ mobility in a highly buffered solution has significant consequences for H^+^ transporting proteins in the cell membrane. Computational models of proton diffusion revealed that H^+^ transporters like MCTs show an apparent turnover rate that exceeds the maximum supply capacity for protons by diffusion [124,125]. That means that MCTs operate at an rate that is way above the rate of H^+^ (buffer) diffusion to or away from the transport pore [124,125]. This paradoxical observation implies that H^+^ transporters need to receive protons not only directly from the bulk solution, but from an additional, intermediate “harvesting” compartment [125]. Such H^+^ harvesting antennae have been described in cytochrome c oxidase or bacteriorhodopsin [126,127]. In short, these proton harvesters are composed of a cluster of acidic glutamate and aspartate residues that function as “proton collectors” and histidines that function as “proton retainers”. Thereby, the antenna enhances the protonation rate of functional groups and creates a “H^+^ reservoir” on the protein surface [128]. Since MCTs lack such a proton collecting mechanism at their surface, it was suggested, that CAs might function as external proton antennae for the transporter which collect or distribute H^+^ from/to protonatable residues in the immediate vicinity of the transporter [129]. The rapid transfer of H^+^ between transporter and surrounding protonatable residues in the immediate vicinity of the transporter would overcome slow H^+^ diffusion in the cytosol or extracellular space and prevent the formation of local H^+^ microdomains (regional depletion or build-up of H^+^ around the transporter pore) (Figure 2). Indeed, fluorometric H^+^ imaging in *Xenopus* oocytes demonstrated that protons, which enter the cell via MCTs at a focal spot, travel longer distances along the inner face of the plasma membrane when CAII is present in cell [130].

CAIX is comprised of a catalytic domain, which is tethered to the extracellular face of the cell membrane via a single transmembrane domain, and a short intracellular C-terminal tail. Furthermore, CAIX features an N-terminal proteoglycan-like (PG) domain, which is unique to CAIX and plays a role in the formation of focal adhesion contacts during cell migration [131,132]. The 59 amino acid long PG domain of human CAIX contains 18 glutamate and 8 aspartate residues. These 26 acidic amino acids have been suggested to function as an intramolecular H^+^ buffer for the CAIX when operating in an acidic environment [133]. Coexpression of MCT1/4 with a truncated form of CAIX, lacking the PG domain, did not facilitate MCT transport activity in *Xenopus* oocytes [134]. These results let to the theory, that CAIX might facilitate the aspartate and glutamate residues in the PG domain to move protons between the MCT transporter pore and surrounding protonatable residues at or near the extracellular side (Figure 2). In line with these results, former studies on the intracellular CAII revealed that CAII, which binds to the C-terminal tail of MCT1 and MCT4 [95,135], facilitates the two acidic residues Glu69 and Asp72 to shuttle protons between the MCT transporter pore and surrounding protonatable residues [136]. However, the identity of the surrounding protonatable residues, which could donate or receive H^+^ to/from the CA is still unknown. This lack of data has to be attributed to the almost infinite number of possible H^+^ donors and acceptors within a living cell. In line with the theory that the PG domain functions as a proton antenna application of an antibody against the CAIX PG domain, but not against the catalytic domain, significantly decreased CAIX-mediated increase in MCT transport activity in *Xenopus* oocytes as well as in MCF-7 and MDA-MB-231 cells [134]. In hypoxic breast cancer cells, application of the antibody against the PG domain did not only decrease lactate flux, but also inhibited cell proliferation, as did knockdown of CAIX with siRNA [115,134]. In contrast, inhibition of CAIX catalytic activity, or application of an antibody against the CAIX catalytic domain, did not alter proliferation of cancer cells [115,134]. Taken together, the data indicate that CAIX forms a transport metabolon with MCT1/4 via their chaperon CD147 in breast cancer cells. Formation of the complex is mediated by hydrogen bonding between Glu73 in the CD147-Ig1 domain and His200 in CAIX. Aspartate and glutamate residues in the PG domain of CAIX facilitate the movement of protons between the transporter pore and surrounding protonatable residues at or near the outer membrane surface. Thereby, CAIX functions as a proton antenna for the transporter to facilitate proton-coupled lactate efflux from hypoxic cancer cells to maintain glycolysis and cell proliferation (Figure 2).

Even though CAIX is considered the most important CA isoform for pH regulation in cancer cells, other carbonic anhydrases can also form transport metabolons in those cells. Knockdown, but not chemical inhibition, of intracellular CAII resulted in a decrease in lactate transport capacity and cell proliferation in both normoxic and hypoxic MCF-7 cells [136]. Interestingly, the effect of CAII knockdown was more pronounced in hypoxic cells (expressing CAIX) than in their normoxic counterparts. This suggests that intracellular CAII and extracellular CAIX may cooperate to facilitate lactate flux across the cell membrane (Figure 2). Such a “push and pull principle” was also observed for MCT1/4, CAII and CAIV in *Xenopus* oocytes [139]. The basic structure and function of the MCT-CAII transport metabolon seems to resemble the MCT-CD147-CAIX complex. CAII was shown to bind directly to the C-terminal tail of MCT1 and MCT4. Binding is mediated by CAII-His64 (which also functions as central residue of the CAII intramolecular H^+^ shuttle) and a cluster of three glutamic acid residues in the MCT C-terminal tail (E489EE in MCT1 [94], and E431EE in MCT4 [135]). Interestingly, His64 is not involved in proton transfer between enzyme and transporter. Proton transfer is instead mediated by Glu69 and Asp72, which are located at the surface of the CAII protein and have been suggested to form a proton-collecting antenna for the enzyme [136]. By this proton-shuttling mechanism, hypoxic cancer cells, which produce high amounts of lactate and protons, can ensure constant extrusion of both ions to protect themselves from larger intracellular acidification and keep up their high glycolytic rate.

Besides the facilitation of H^+^-coupled lactate export from cancer cells via MCTs, CAIX could also drive glycolysis by regulation of different signaling pathways under hypoxia. In a recent study, silencing of CAIX in hypoxic breast cancer cells increased the level of the regulatory microRNA *let-7* and decreased the level of the RNA-binding protein LIN28 [140]. Aberrant expression of Lin28 and its downstream target *let-7* had previously been shown to facilitate aerobic glycolysis in cancer cells by activation of pyruvate dehydrogenase kinase 1 (PDK1) [141,142]. PDK1 inactivates the pyruvate dehydrogenase complex (PDH) and thereby blocks the conversion of pyruvate to acetyl-CoA, inhibiting the citric acid cycle and promoting glycolysis. In line with this, knockdown of CAIX resulted in decreased lactate production in cancer cells [140]. Interestingly, silencing of CAIX did also decrease the expression of NF-κB, which functions as a direct activator of LIN28. Furthermore, incubation of the cells at low pH_e_ values resulted in increased expression of NF-κB, while pharmacological inhibition of CAIX catalytic activity decreased NF-κB expression, indicating that CAIX drives expression of NF-κB, and thereby glycolytic activity, through its pH regulatory function [140]. In line with these findings, transient knockdown of CAIX in hypoxic HeLa cells decreases the level of the glycolytic key enzymes, phosphoglycerate kinase, enolase, and fructose-bisphosphate aldolase, as well as the enzyme lactate dehydrogenase A (LDH-A), which converts pyruvate into lactate [143]. The decrease in LDH-A activity directly correlated with the CAIX-knockdown efficacy, indicating functional coupling between the two proteins. Furthermore, both knockdown of CAIX and overexpression of a catalytically inactive CAIX isoform reduced pH_i_, which resulted in attenuated glycolytic flux, reduced export of lactate and protons, and ultimately reduced cell proliferation. Interestingly, application of α-ketobutyrate (α-KB), which serves as an alternative substrate for LDH-A, reversed the drop in pH_i_ in CAIX knockdown cells by increased proton secretion, and rescued glycolytic flux and cell proliferation. These data indicate that CAIX stabilizes pH_i_ not only by production of HCO_3_^−^ ions and facilitated extrusion of lactate and protons via MCTs, but also by maximizing glycolytic flux through increased LHD-A activity [143].

## 6. Targeting Transport Metabolons for Cancer Therapy

Targeting the acid/base regulatory machinery of cancer cells appears to be a promising tool for cancer therapy. Disruption of tumor pH homeostasis could either affect cancer cell function directly or could render the cells more susceptible for conventional therapy. Indeed, several acid/base transporters have been considered as potential targets for the treatment of cancer. For example, inhibition of NHE1 transport activity with chemical inhibitors like cariporide, EIPA, DMA or amiloride was shown to sensitize chemo-resistant cancer cells for chemotherapeutic drugs like doxorubicin [144,145], Imatinib [146] or paclitaxel [147]. However, cariporide failed in phase 3 clinical trial as therapeutic drug for the treatment of myocardial infarction, due to the occurrence of severe side effects [148,149]. This demonstrates that direct inhibition of acid/base transport for cancer therapy could lead to complications due to the widespread expression of these transport proteins in healthy tissue. 

Since expression of CAIX is mostly restricted to cancer cells, targeting of CAIX transport metabolons might pose a more specific impact on cancer cells than targeting of the transporters directly. Bicarbonate transport metabolons per se have not been targeted for tumor therapy so far, but since these metabolons require CAIX catalytic activity, it can be assumed that conventional CA inhibitors also target transport metabolons. CA inhibitors are already used in the treatment of various diseases, including glaucoma [150,151], epilepsy [152,153,154] and high-altitude sickness [155,156]. Furthermore, CAs have been suggested to serve as therapeutic targets in the treatment of neuropathic pain [157] and obesity [158]. In solid tumors, the acidic tumor environment, which is created by CAIX catalytic activity, provides a potential target for cancer therapy, since it is a unique feature for solid tumors and a common phenotype of a wide spectrum of cancer types [54]. Indeed, various preclinical studies have shown that CA inhibitors like acetazolamide derivates, glycosyl coumarins, or the ureido-substituted benzenesulfonamide SLC-0111 can inhibit tumor growth, formation of metastasis, and reverse malignancy in cultured cancer cells, in spheroids and in tumor xenografts [159,160,161,162]. However, inhibition of CAIX might be most efficient in a smart combination therapy [163]. For example, inhibition of CAIX activity with acetazolamide rendered hypoxic HT29 colon carcinoma cells more susceptible to the chemotherapeutical drug doxorubicin [164].

Although inhibition of CAIX catalytic activity by conventional CA inhibitors might also target bicarbonate transport metabolons, those inhibitors would not target the MCT-CD147-CAIX transport metabolon, since CAIX-dependent facilitation of MCT activity is independent of CA catalytic activity. Indeed, inhibition of CAIX catalytic activity with three CAIX-specific ureido-substituted benzene sulfonamides did not alter glycolytic flux (glycoPER) in pseudohypoxic UFH-001 breast cancer cells, while knockout of CAIX reduced glycoPER [116]. In line with these findings, the same group could show that inhibition of CAIX activity with SLC-149, a patented, imidazole-substituted benzenesulfonamide inhibitor, which is currently in preclinical trial stage [165], did not affect the growth of cultivated breast cancer cells [118]. In contrast, application of an antibody against the CAIX-PG domain, which was suggested to function as proton antenna for the metabolon, resulted in a decrease in lactate transport capacity, cell proliferation and migration in hypoxic MCF-7 and MDA-MB-231 cells [123]. In line with this, application of an antibody against the CD147 Ig1 domain, close to the CAIX binding site, resulted in a decrease in lactate transport capacity and cell proliferation in hypoxic MCF-7 and MDA-MB-231 cells. The antibody displaces CAIX from the MCT-CD147 complex and leads to disruption of the transport metabolon [117]. These experiments provide a proof of concept that targeting of the MCT-CD147-CAIX transport metabolon, either by interference with the CAIX antenna function or by disruption of the protein complex, can provide a useful tool for future tumor therapy.

## 7. Conclusions

Mechanisms of pH regulation belong to a number of unique adaptations of cancer cells to allow tumor tissue to grow and migrate in spite of unfavorable conditions such as hypoxia and the production of large amounts of acid. In order to avoid severe acidosis, expression of CA isoform IX in tumor tissue, in addition to other intra- and extracellular CA isoforms, supports proton translocation. In particular, the formation of transport metabolons with acid/base and metabolite transporters, whereby CAs operate as proton antennae, can promote survival and growth of tumors. Such transport metabolons may well serve as specific targets for therapeutic interventions to derange proton transport and pH regulation in cancer cells. 

## Data Availability

No new data were created or analyzed in this review. Data sharing is not applicable to this article.

## Abbreviations

α-KB	α-ketobutyrate
AE	Cl^−^/HCO_3_^−^ anion exchanger
CA	carbonic anhydrase
DFODMA	desferrioxamine5-(N,N- dimethyl) amiloride
glycoPER	glycolytic Proton Efflux Rate
EIPA	5-(N-Ethyl-N-isopropyl)amiloride
EZA	6-Ethoxy-2-benzothiazolesulfonamide
FRET	Förster resonance energy transfer
Ig	Immunoglobulin
LDH-A	lactate dehydrogenase isoform A
MAPK	mitogen-activated protein kinase
MCT	monocarboxylate transporter
mTORC1	mammalian target of rapamycin complex 1
NBC	Na^+^/HCO_3_^−^ cotranspoter
NCX	Na^+^/Ca^2+^ exchanger
NF-κB	nuclear factor ‘kappa-light-chain-enhancer’ of activated B-cells
NHE	Na^+^/H^+^ exchanger
PDAC	pancreatic ductal adenocarcinoma
PDH	pyruvate dehydrogenase
PDK	pyruvate dehydrogenase kinase
PG	proteoglycan-like
PLA	proximity ligation assay
TCA	tricarboxylic acid cycle
VHL	von Hippel–Lindau tumor suppressor
